# Human Monocytes Undergo Excessive Apoptosis following Temozolomide Activating the ATM/ATR Pathway While Dendritic Cells and Macrophages Are Resistant

**DOI:** 10.1371/journal.pone.0039956

**Published:** 2012-06-29

**Authors:** Martina Bauer, Michael Goldstein, Daniel Heylmann, Bernd Kaina

**Affiliations:** Department of Toxicology, University Medical Center, Mainz, Germany; Hannover Medical University (MHH), Germany

## Abstract

Immunodeficiency is a severe therapy-limiting side effect of anticancer chemotherapy resulting from sensitivity of immunocompetent cells to DNA damaging agents. A central role in the immune system is played by monocytes that differentiate into macrophages and dendritic cells (DCs). In this study we compared human monocytes isolated from peripheral blood and cytokine matured macrophages and DCs derived from them and assessed the mechanism of toxicity of the DNA methylating anticancer drug temozolomide (TMZ) in these cell populations. We observed that monocytes, but not DCs and macrophages, were highly sensitive to the killing effect of TMZ. Studies on DNA damage and repair revealed that the initial DNA incision was efficient in monocytes while the re-ligation step of base excision repair (BER) can not be accomplished, resulting in an accumulation of DNA single-strand breaks (SSBs). Furthermore, monocytes accumulated DNA double-strand breaks (DSBs) following TMZ treatment, while DCs and macrophages were able to repair DSBs. Monocytes lack the DNA repair proteins XRCC1, ligase IIIα and PARP-1 whose expression is restored during differentiation into macrophages and DCs following treatment with GM-CSF and GM-CSF plus IL-4, respectively. These proteins play a key role both in BER and DSB repair by B-NHEJ, which explains the accumulation of DNA breaks in monocytes following TMZ treatment. Although TMZ provoked an upregulation of XRCC1 and ligase IIIα, BER was not enhanced likely because PARP-1 was not upregulated. Accordingly, inhibition of PARP-1 did not sensitize monocytes, but monocyte-derived DCs in which strong PARP activation was observed. TMZ induced in monocytes the DNA damage response pathways ATM-Chk2 and ATR-Chk1 resulting in p53 activation. Finally, upon activation of the Fas-receptor and the mitochondrial pathway apoptosis was executed in a caspase-dependent manner. The downregulation of DNA repair in monocytes, resulting in their selective killing by TMZ, might impact on the immune response during cancer chemotherapy.

## Introduction

Immunosuppression is one of the most severe side effects of chemotherapy endangering lives of patients who undergo medical cancer treatment. In general, the high proliferation rate of the immune response progenitor cells is considered accountable for their sensitivity to DNA damaging agents that are used for cancer treatment. Surprisingly, little attention has been paid yet to the toxicity of chemotherapeutic drugs in mature immune response cells. Originating from bone marrow precursor cells mature monocytes enter the blood stream where they circulate for several days [1]. After entering the tissue they differentiate into DCs and macrophages, both of which play an important role in the immune response.

In the course of the current research we investigated the mechanism of cytotoxicity of the chemotherapeutic anticancer drug temozolomide (TMZ, Temodar) in human monocytes freshly isolated from peripheral blood. Methylating agents, including TMZ, induce a variety of N- and O-DNA alkylations with N7-methylguanine to be the most frequent one [2]. O^6^-methylguanine is a minor adduct, which is repaired by O^6^-methylguanine-DNA methyltransferase (MGMT) [3]. If this repair mechanism fails O^6^-methylguanine results in the formation of toxic DSBs due to faulty mismatch repair during proliferation [4]. On the other hand, N7-methylguanine and other N-methylpurines like the replication blocking N3-methyladenine are repaired by base excision repair (BER) [5]. In a previous work we reported that human monocytes express the BER factors XRCC1 and ligase IIIα at a low, nearly undetectable level, which was restored during the *ex vivo* differentiation of monocytes to dendritic cells (DCs) [6], suggesting a defect of BER in monocytes. Indeed, monocytes were hypersensitive to DNA methylating agents, while DCs derived from them were not [6].

As mentioned above, non-toxic DNA lesions such as DNA alkylation adducts can be converted into DNA single-strand (SSB) and double-strand breaks (DDB) resulting in cytotoxicity. SSB are detected by the ATR kinase, while DSB activate the ATM kinase. A variety of signaling pathways is activated in turn, resulting in cell cycle arrest and apoptosis, which in many cases is p53-dependent (for review see [7]). Here, we extend our previous observation by showing that monocytes strongly respond to TMZ. They do not express PARP-1, which is another BER, SSB and DSB repair factor [8], [9]. Similar to XRCC1 and ligase IIIα, PARP-1 expression is upregulated during differentiation of monocytes into DCs and macrophages. We further demonstrate that monocytes can initiate BER by incising the DNA. However, lack in XRCC1, PARP-1 and ligase IIIα prevents subsequent DNA re-ligation resulting in accumulation of SSBs. Following TMZ treatment the inability to complete DNA repair finally results in an accumulation of DSBs in monocytes, but not in DCs and macrophages. Our data bear important clinical implications, suggesting that mature monocytes may be specifically killed during TMZ based cancer therapy, whereas DCs and macrophages might be protected.

## Results

**Figure 1:**
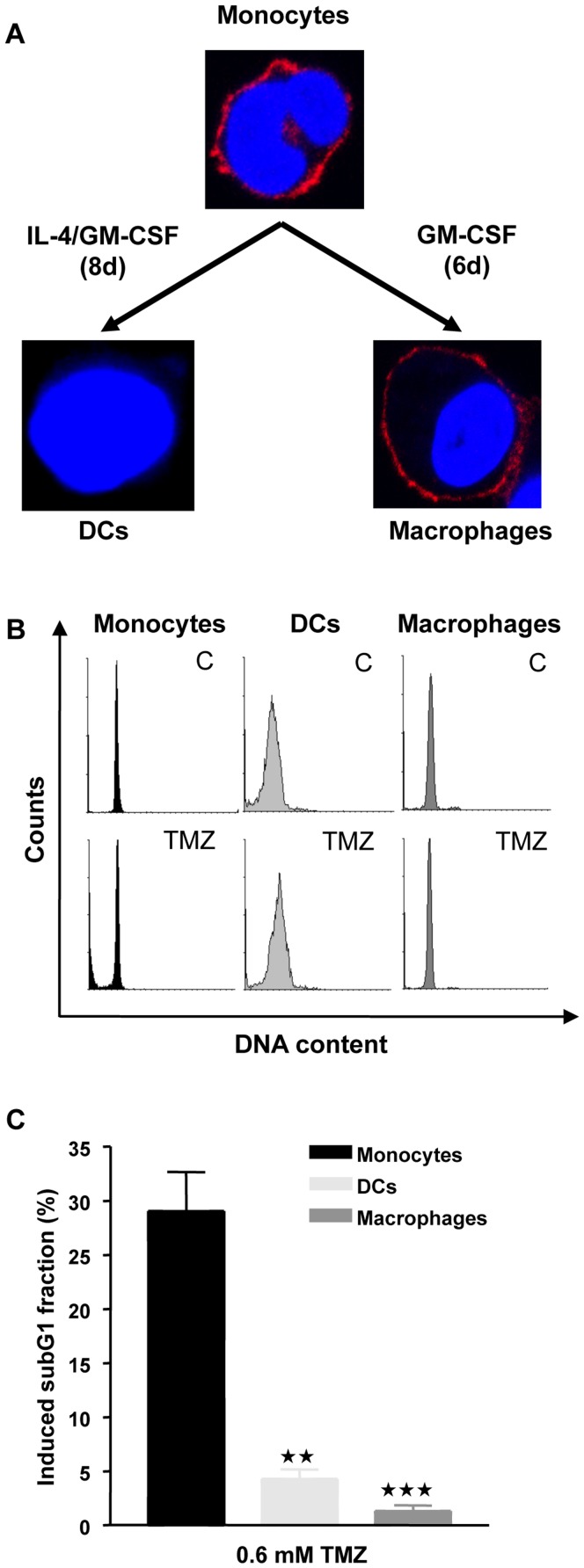
Differentiation of monocytes into DCs and macrophages and their killing response. (A) Images of monocytes and DCs and macrophages derived from them by cytokine stimulated maturation. Blue, nuclear staining with ToPro3; red staining on monocytes and macrophages, CD14 surface marker, which is not expressed on macrophages. (B) Representative histograms of monocytes, DCs and macrophages stained with propidium iodide and measured by flow cytometry. C, non-treated; TMZ, treated with 0.6 mM TMZ and measured 72 h after treatment. (C) Analysis of apoptosis 72 h following 0.6 mM TMZ treatment by quantification of the subG1 fraction of cells by flow cytometry. (**p<0.01, ***p<0.001, *t-test* comparing monocytes with DCs and macrophages).

In order to study the TMZ sensitivity and DNA damage response in human monocytes and their derivatives, macrophages and immature DCs, monocytes were isolated from peripheral blood of healthy donors and either left untreated or treated with IL-4 and GM-CSF or GM-CSF alone in order to induce the differentiation of DCs or macrophages, respectively (Fig. 1A showing the typical shape and surface staining of monocytes and macrophages with CD14). The cell populations from each preparation were characterized by flow cytometry (see Material and Methods). It is noteworthy that, except for small populations, mature monocytes, DCs and macrophages do not proliferate *in vivo* [10]. In fact, as revealed by flow cytometry (Fig. 1B), all three cell types were arrested in G1 and maintained *in vitro* in a non-proliferative state. Treatment with the methylating anticancer drug TMZ resulted in a significant induction of apoptosis in monocytes, while DCs and macrophages were resistant to this agent (Fig. 1C).

**Figure 2:**
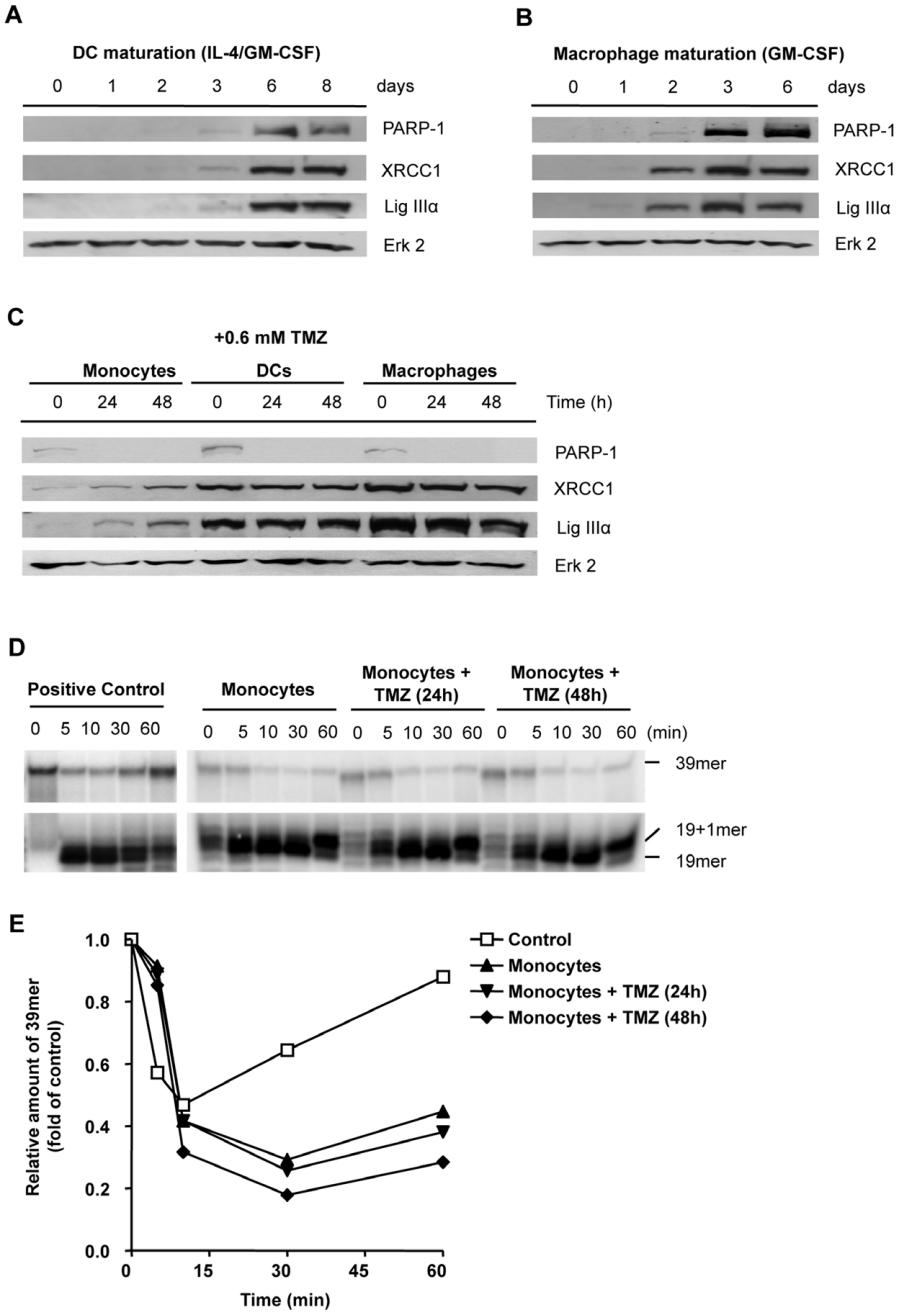
Expression of BER proteins in monocytes and follow-up during differentiation of monocytes in DCs and macrophages and BER activity in monocytes. Expression of PARP-1, XRCC1 and ligase IIIα during maturation of monocytes into DCs (A) and macrophages (B) analyzed by immunoblots. (C) Expression of PARP-1, XRCC1 and ligase IIIα in monocytes, DCs and macrophages without treatment and 24 and 48 h following treatment with 0.6 mM TMZ. (D) BER assay in untreated and TMZ treated monocytes 24 and 48 h post-treatment. The 39mer fragment represents the full-length oligonucleotide. The 19mer and the 19+1 fragment are products of the initial incision and processing, respectively. Restoration of the 39mer fragment represents the re-ligation step. For positive, repair competent, control we used extracts of DCs. (E) Quantification of the full-length fragment shown in (D). The relative amount of the 39mer is shown as a function of time following TMZ.

DNA methylation following TMZ exposure yields different lesions with N7-methylguanine to be the most frequent one to appear [2]. Although O^6^-methylguanine was shown to be responsible for the toxicity of methylating agents in proliferating cells [4], this lesion is unlikely to be important in our cellular system since the cells do not proliferate. Also, monocytes express a high level of MGMT compared to macrophages and DCs and are therefore able to remove O^6^-methylguanine from DNA [11]. N7-MeG, N3-MeG and the toxic lesion N3-MeA are repaired by BER. This prompted us to determine the expression level of BER enzymes and we found, similar to our previous observation [6], that monocytes express XRCC1 and ligase IIIα at a very low (non-detectable) level (Fig. 2A,B). Expression of these BER factors was evoked, however, following cytokine treatment and was completely restored on day 6 (Fig. 2A) and day 3 (Fig. 2B) during maturation of monocytes into DCs and macrophages, respectively. We also observed that monocytes lack PARP-1, an important factor of BER and SSB repair, and that expression of PARP-1 was restored concomitant with XRCC1 and ligase IIIα during the maturation of monocytes to DCs and macrophages (Fig. 2A,B).

Next we wished to determine whether treatment with TMZ has an effect on the expression of the repair proteins. TMZ treatment resulted in decreased PARP-1 levels in DCs and macrophages. Surprisingly, it also resulted in an increase in the level of XRCC1 and ligase IIIα in monocytes (Fig. 2C). Thus it appears that TMZ treatment provokes upregulation of XRCC1 and ligase IIIα, but not PARP1 in monocytes. This prompted us to study the BER activity in monocytes following genotoxic stress by TMZ. Without TMZ treatment, monocytes were unable to restitute the cleaved oligonucleotide (Fig. 2D). Despite the increase in XRCC1 and ligase IIIα level following TMZ treatment, the BER activity in monocytes was not restored (Fig. 2D). Thus, the initial incision of the lesion (yielding the 19mer fragment) and the following processing (yielding the 19+1mer fragment) was highly efficient in monocytes, while the XRCC1/ligase IIIα-dependent re-ligation step (resulting in the restoration of the 39mer) was abrogated (Fig. 2D and 2E for quantification). The accumulation of the 19+1mer fragment reflected the increased level of SSBs in monocytes due to incomplete BER.

**Figure 3:**
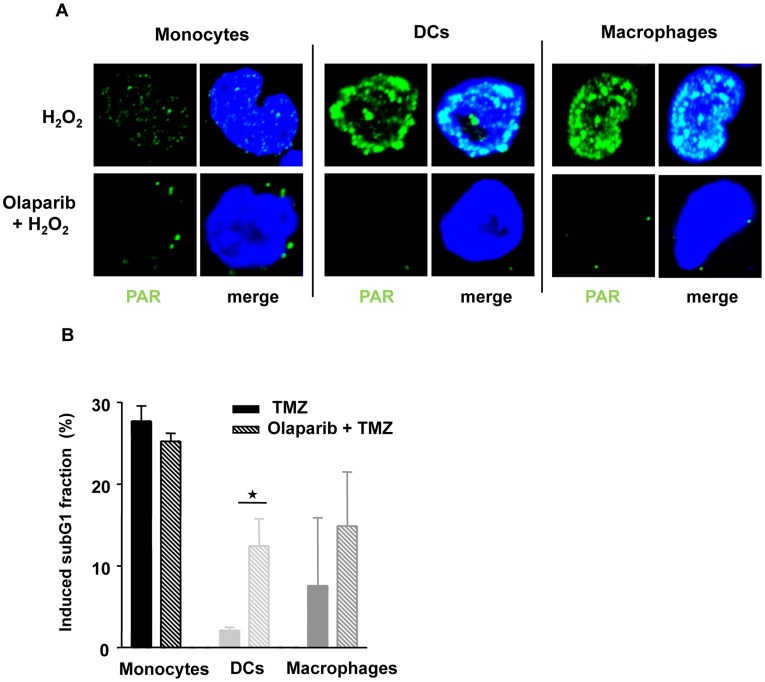
PARP activation and effect of PARP inhibition in monocytes, macrophages and DCs. (A) Cells were treated with hydrogen peroxide (10 mM for 10 min) or olaparib (0.5 µM) 1 h prior to hydrogen peroxide (10 mM, 10 min), fixed and stained with anti-poly(ADP)ribose (PAR) antibody. Green, anti-PAR; blue, nuclear staining with ToPro3. (B) Cells were treated with temozolomide (0.6 mM) or olaparib (0.5 µM) 1 h prior to temozolomide and apoptosis was measured by subG1 flow cytometry 72 h later. Basal levels were subtracted and the induced levels are shown. Data are the mean of three independent experiments +/−S.D.

The lack of PARP-1 in monocytes let us to predict that monocytes are unable to generate poly(ADP)ribose following genotoxic stress. This is indeed the case as monocytes did not display PAR staining while DCs and macrophages were heavily PAR stained upon treatment with hydrogen peroxide, which induces oxidative DNA damage (Fig. 3A). The PARP-1 inhibitor olaparib [12] abolished the formation of PAR nearly to completion (Fig. 3A). As expected, olaparib had no effect on the killing response of monocytes treated with TMZ, while it enhanced cell death in DCs and macrophages (Fig. 3B). We should note that the effect of olaparib in DCs and macrophages on TMZ-induced cell death was not dramatic and the cells did not reach the sensitivity level of monocytes, which is explained by the finding that monocytes lack in addition to PARP-1 other BER proteins that exacerbate the deficiency of PARP-1. Overall, the data supports the notion that the lack of PARP-1 together with XRCC1, ligase IIIα and DNA-PKcs is critically involved in the hypersensitivity of monocytes to TMZ.

**Figure 4:**
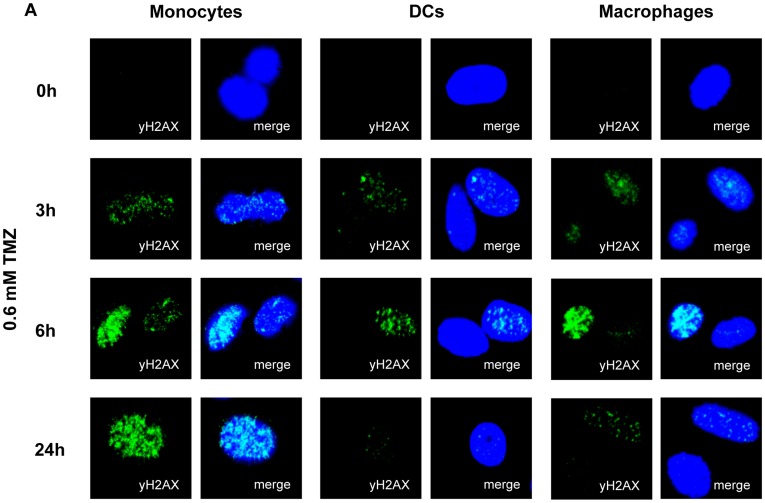
Accumulation of DSBs in monocytes, but not in DCs and macrophages after TMZ treatment. Immunostaining of γH2AX foci at indicated time points following treatment with 0.6 mM TMZ in monocytes, DCs and macrophages. Blue, nuclear staining with ToPro3; green, phospho-H2AX staining with anti-γH2AX.

Following DNA methylation, SSBs can be converted to toxic DSBs during the S phase [13]. Monocytes, however, are non-proliferating cells. In these cells an induction of a large amount of SSBs can result in DSB formation if two SSBs, one on each DNA strand, face each other. Since it is reasonable to posit that the cytotoxicity observed in monocytes originates from DSBs we determined the formation of γH2AX foci that reflects DSB formation [14]. A similar formation of γH2AX foci was observed in monocytes, DCs and macrophages 3 h after TMZ treatment (Fig. 4) indicating that DSBs are formed in all three cell types following DNA methylation. However, while the resolution of γH2AX foci in DCs and macrophages indicated that repair of the DNA lesions occurred in these cells, DSBs remained in monocytes for as long as 24 h post-treatment (Fig. 4), indicating a defect in DSB repair.

**Figure 5:**
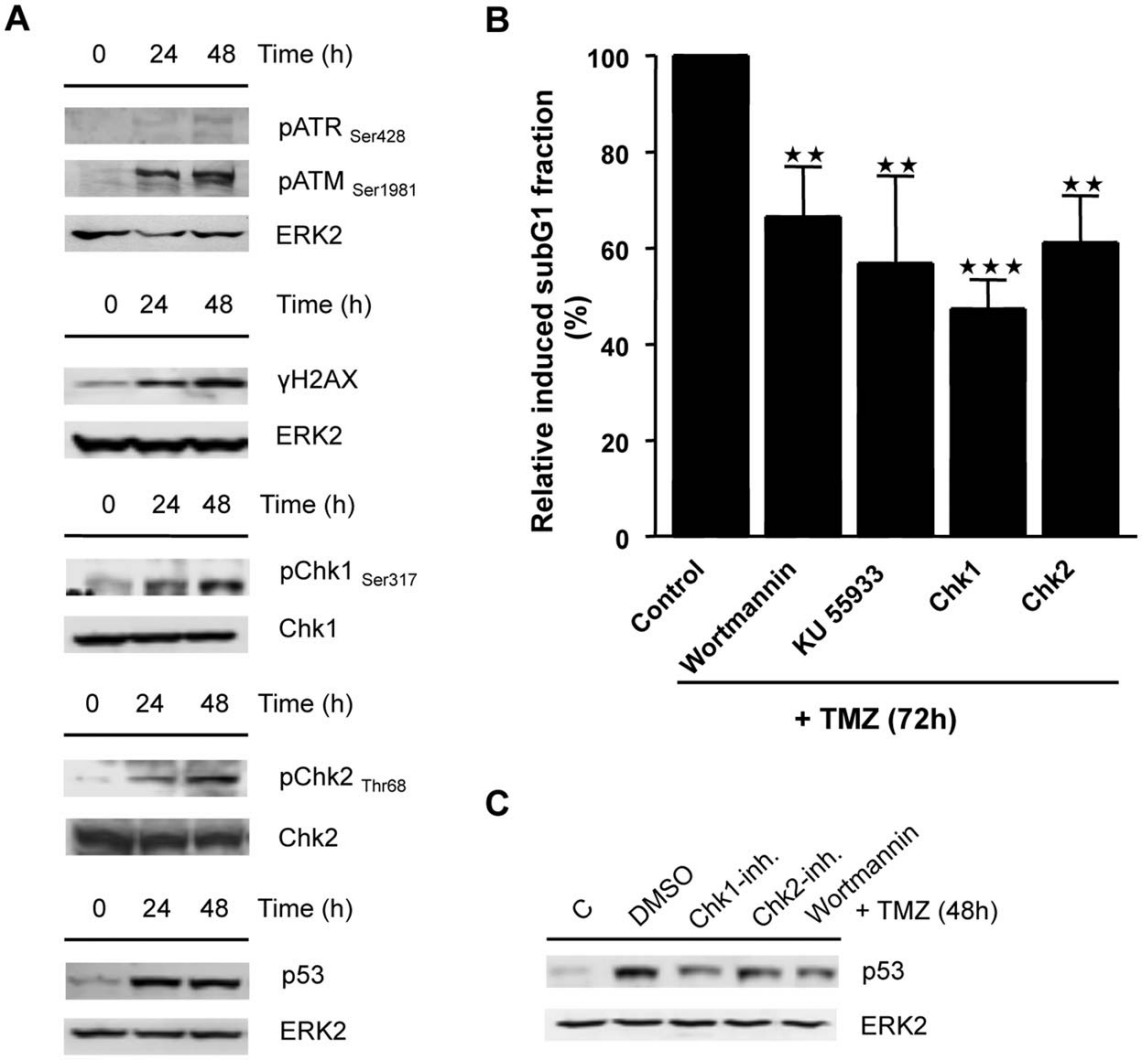
Activation of ATM, ATR, p-H2AX, Chk1, Chk2 and p53 in monocytes. (A) Western Blot analysis of DDR proteins and p53 in monocytes not treated (zero time) and treated with 0.6 mM TMZ. (B) Quantification of the subG1 fraction in monocytes co-treated with 0.6 mM TMZ and the indicated kinase inhibitors for 72 h. (C) Western Blot analysis of p53 activation in monocytes co-treated with 0.6 mM TMZ and kinase inhibitors as indicated for 48 h. Cells were pretreated with 10 µM wortmanin, 10 µM Ku55933, 10 µM Chk1 and 10 µM Chk2 inhibitor for 1 h prior to TMZ addition. Cells were post-treated with 10 µM Ku55933, 10 µM Chk1 and 10 µM Chk2 inhibitor every 24 h following TMZ treatment.

Next, we addressed the question of how apoptotic cell death is executed in monocytes following TMZ-induced DNA lesions. DSBs activate the ATM kinase, which in turn can activate p53 either directly by phosphorylation or via activation of the Chk2 kinase [7]. SSBs activate the ATR kinase that phosphorylates p53 either directly or via Chk1 activation [7]. In Fig. 5A it is shown that in monocytes following TMZ treatment both ATM and ATR are activated, which is reflected by autophosphorylation of these kinases and by consecutive phosphorylation of the histone H2AX (forming γH2AX). We also observed activation of Chk1 and Chk2 and a strong increase in p53 protein level (Fig. 5A). Applying a number of specific small molecule inhibitors of ATM, ATR, Chk1 and Chk2 we show that all these DNA damage response (DDR) factors contribute to TMZ-induced apoptosis in monocytes since apoptosis was reduced following their cotreatment with TMZ (Fig. 5B). Inhibition of Chk1, Chk2 and PI-3 kinases attenuated the level of induction of p53 protein (Fig. 5C) indicating that these factors act upstream of p53.

**Figure 6:**
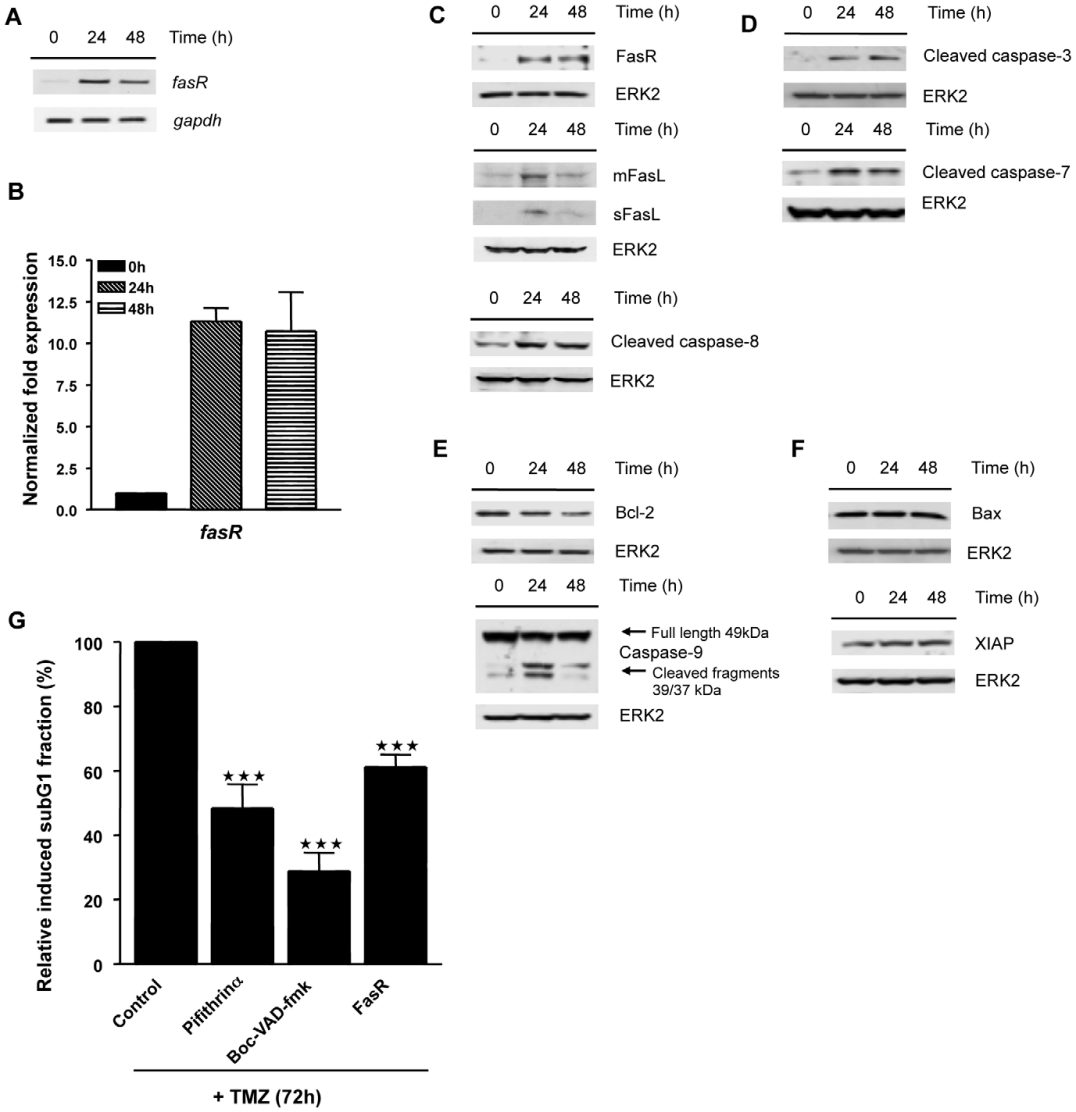
Mitochondrial and FasR pathway activation in monocytes resulting in caspase dependent apoptosis. (A) Representative image of semiquantitative RT-PCR analysis of FasR mRNA expression in monocytes treated with 0.6 mM TMZ. (B) Quantitative RT-PCR analysis of FasR mRNA expression in monocytes treated with 0.6 mM TMZ. (C, D, E, F) Western Blot analysis of Fas receptor, membrane bound Fas ligand and cleaved caspase-8 (C) activated caspase-3 and -7 (D) Bcl-2 and activated caspase-9 (E) and BAX and XIAP (F) in monocytes treated with 0.6 mM TMZ. (G) Quantification of the subG1 fraction in monocytes co-treated with 0.6 mM TMZ and indicated inhibitors or antibody for 72 h. Cells were pretreated with 30 µM pifithrin-α, 50 µM Boc-VAD-fmk and 1 µg/ml anti FasR antibody for 1 h prior to the addition of TMZ and post-treated with 15 µM pifithrin-α, 25 µM Boc-VAD-fmk and 0.5 µg/ml anti FasR antibody every 24 h following TMZ treatment.

We went on to investigate which pathway signals apoptosis in monocytes downstream of p53. Following TMZ treatment we observed an increase in the death receptor Fas (CD95, Apo-1). FasR upregulation following TMZ was observed on mRNA level (Fig. 6A for semi-quantitative PCR and Fig. 6B for qRT-PCR), which was confirmed on the level of the protein (Fig. 6C). At the same time we observed an increase in the level of membrane-bound (mFasL) and soluble (sFasL) Fas ligand and cleavage of caspase-8 (Fig. 6C). The data strongly suggest the involvement of the exogenous Fas-driven pathway in TMZ-induced monocyte toxicity. Downstream we observed activation of the executing caspases -3 and -7 (Fig. 6D). There was also Bcl-2 decline and caspase-9 activation (Fig. 6E) indicating the mitochondrial pathway to be involved as well. Bax and XIAP were not changed in expression (Fig. 6F).

The involvement of caspases and p53 in TMZ-induced apoptosis in human primary monocytes was confirmed by inhibitor experiments showing that the transcriptional inhibitor of p53, pifithrin-α, as well as the pan-caspase inhibitor Boc-VAD-fmk and an antagonizing Fas receptor antibody significantly attenuated the apoptotic response (Fig. 6G). Overall, the data showed that TMZ induces the ATM/ATR/p53 response in human monocytes that results downstream in activation of the endogenous and exogenous apoptosis pathway.

## Discussion

Cancer patients who undergo chemotherapy frequently suffer from immunosuppression, making it one of the most important dose-limiting side effects. The reason for this is believed to be that chemotherapeutic drugs that target DNA require DNA replication in order to become cytotoxic [15] and, therefore, cells are most sensitive towards most types of DNA lesions in the S phase of the cell cycle. Based on this, a current paradigm states that highly proliferative tissues such as the tumor itself and bone marrow are most responsive to chemotherapy. Although immune response precursor cells are known to be very susceptible, which is thought to be due to hematopoetic stem cell toxicity [16], the majority of mature immune response cells does not proliferate and might thus be considered resistant to chemotherapy. Challenging this hypothesis, we investigated the mechanism of cytotoxicity of the chemotherapeutic drug TMZ, which is representative for methylating agents and used in glioma and malignant melanoma therapy, in non-proliferating human monocytes freshly isolated from peripheral blood, and DCs and macrophages derived from them by cytokine maturation.

Here, we report that primary monocytes are highly sensitive to TMZ while DCs and macrophages (derived in each experiment from the same donor) are resistant. TMZ is a methylating agent inducing N- and O-alkylations in the DNA. Although N7-methylguanine is the most frequent lesion induced by methylating agents [2] O^6^-methylguanine is responsible for the cytotoxicity in proliferating cells due to faulty MMR and replication-dependent DSB formation [17], [18]. Since CD14^+^ monocytes isolated from peripheral bloood, and DCs and macrophages derived from them (defined by the surface markers CD3, CD19, CD14, CD80, CD86, [19]) were cultured under conditions that do not allow proliferation, a significant contribution of O^6^-methylguanine to the toxicity in these cells is unlikely. Accordingly, inhibition of MGMT, which is highly expressed in monocytes, had no effect on the cytotoxicity in these cells following treatment with the methylating mutagen MNNG [6]. Therefore, we focused on N7-methylguanine and N3-methyladenine as potentially toxic lesions. Since these adducts are repaired by BER that requires XRCC1, ligase IIIα and PARP-1, we conclude that the hypersensitivity to TMZ observed in monocytes results from the lack of expression of these BER factors. These repair proteins are upregulated in DCs and macrophages and therefore DNA repair, i.e. the re-ligation step of BER, is restored upon maturation. Interestingly, following genotoxic stress by TMZ treatment the level of XRCC1 and ligase IIIα increased in monocytes, which extends a previous finding showing that XRCC1 is upregulated in *in vitro* cultured monocyte-like cells following methyl methanesulfonate treatment [20]. Nonetheless, these TMZ-stimulated alterations in protein expression did not improve the DNA break re-ligation efficiency in monocytes following TMZ treatment. The reason lies likely in the lack of PARP-1, which was not upregulated following TMZ treatment. PARP-1 is functionally active in macrophages and DCs, but not monocytes, as shown by extensive PAR formation in macrophages and DCs following genotoxic stress. Inhibition of PARP-1 by olaparib sensitized macrophages and DCs to TMZ, but not to the level of monocytes, which is explained by the multiple repair defect in these cells. We should note that DNA-PKcs is also lacking in monocytes [19]. In complementation studies with a bicistronic vector of XRCC1-ligase IIIα we were unable complement the hypersensitive phenotype of monocytes (unpublished data), which is to be expected taking into account the severe DNA repair defect in these cells.

Although in our system DSBs will not arise during DNA replication (as the cells do not replicate), they can emerge when two SSBs are facing each other, which may happen if a large amount of SSBs accumulates in the cell. This appears to be the case as indicated by the time course of formation of γH2AX foci in monocytes, DCs and macrophages after TMZ treatment. While γH2AX foci were resolved in DCs and macrophages 24 h following TMZ treatment, indicating DNA repair, DSBs continued to be present in monocytes. Thus, we conclude that TMZ-induced N-methylpurines, that are subject to removal by N-methylpurine-DNA glycosylase, are converted into SSBs due to incision of DNA by the BER machinery and a fraction of them will result in DSBs as the result of SSB accumulation in overlapping repair patches. It is important to note that monocytes express a normal level of N-methylpurine-DNA glycosylase (MPG alias AAG) and apurinic endonuclease and are thus able to remove N-methylpurines from DNA [6]. While DCs and macrophages can repair the subsequently formed SSBs, monocytes are defective in the ligation of these repair intermediates as this repair step requires XRCC1 and ligase IIIα. Additionally, DSBs formed in overlapping repair patches are not repaired as a subpathway of DSB repair in non-proliferating cells is B-NHEJ, which rests on XRCC1, ligase IIIα and PARP-1 [21]. We should note that homologous recombination does not play a role since the cells were not proliferating. The data leads us to conclude that the hypersensitivity of monocytes to methylating anticancer drugs is a result of a defect in BER and NHEJ.

Regarding the mechanism of cell kill, we found that following TMZ treatment the ATM/ATR-Chk1/Chk2-p53 pathway was activated in monocytes resulting in Fas (CD95, Apo-1) receptor upregulation and caspase-8 activation. We also observed Bcl-2 decline and caspase-9 activation indicating the involvement of the exogenous and endogenous apoptotic pathway, both of which can be activated by DNA damage [22]. The Fas pathway seems to play an important role in the activation of apoptosis following DNA damage in hematopoetic cells as it becomes activated after exposure to mafosfamide, a DNA cross-linking drug [23], oxidative stress [19] and other genotoxic insults [24].

Our results bear implications for cancer treatment. We should note that monocytes are not only TMZ but also ionising radiation (IR) hypersensitive [19], which is important to note since TMZ is applied concomitantly with IR in glioma therapy [25]. Hypersensitivity of monocytes towards TMZ (and IR) could be at least in part responsible for the immunosuppression in patients who undergo chemotherapy, leading to a depletion of monocyte derived macrophages and DCs, which are supposed to play a role in tumor host defense [26], [27], [28]. At the same time our data (this paper and [6], [19]) indicate that immature and mature DCs and macrophages exhibit a significant defense by efficient DNA repair and thus are protected against methylating agents and IR-induced cell death. This is notably important for immuno-vaccination of patients with immature DCs [29], which are derived from monocytes *in vitro* according to the same protocol we used in our experiments [30]. Clinical studies observing monocyte counts in patients receiving TMZ or other methylating drugs such as dacarbazine, procarbazine or streptozotozine would provide further evidence, and these studies are in progress. The finding that both Chk1 and Chk2 inhibitors were able to attenuate the killing response of monocytes following TMZ bears the potential of protecting monocytes from therapy related side effects. These inhibitors are being clinically tested in combination with chemotherapy [31]. Since inhibition of these kinases reduced apoptosis in monocytes we suggest the possibility that inhibitors of Chk1 and Chk2 may protect monocytes during cancer treatment and compensate some of the immunosuppressive effects of chemotherapy. Recently, new approaches have been developed to inhibit DNA damage dependent p53 activation using short, single-strand oligonucleotides that target this 5′-3′-UTR base-pairing region of p53 mRNA and block its translation [32]. Once this approach will be applicable to cancer patients in order to protect bone marrow from side effects of chemotherapy our data suggest that mature monocytes will benefit from this treatment as well.

## Materials and Methods

### Chemicals

Temozolomide (4-methyl-5-oxo-2,3,4,6,8-pentazabicyclo[4.3.0]nona-2,7,9-triene-9-carboxamide; Schering-Plough, Whitehouse Station, NJ) was prepared and used as described previously [33]. Wortmannin, Ku 55933, Isogranulatimide and Chk2 inhibitor II, Pifithrin-α, Boc-VAD-fmk, neutralizing FasR-AB and Protein G were obtained from Calbiochem (Schwalbach, Germany). Wortmannin is an inhibitor of phosphatidylinositol 3-kinase family like ATM and ATR [34]. Ku55933 acts as an inhibitor of ATM kinase [35], [36]. Isogranulatimide is a Chk1 inhibitor [37]. Pifithrin-α inhibits the transcriptional activity of p53 [38].

### Generation of Monocytes, Dendritic Cells and Macrophages from Peripheral Blood Mononuclear Cells (PBMC)

PBMC were isolated by Ficoll-Hypaque density gradient centrifugation from buffy coats of healthy blood donors obtained from the blood bank of University Hospital of Mainz. They were seeded in six-well plates (Corning/Costar, Bodenheim, Germany) at 1–1.5×10^7^ cells/3 ml/well in RPMI 1640 (PAA, Pasching, Austria) containing 1.5% autologous plasma and incubated for 60 min at 37°C, 5% CO_2_. Non-adherent cells were removed and the remaining cells, which represent monocytes [30] were harvested or further cultured in X-VIVO-15 with 800 U/ml GM-CSF (Bayer Healthcare Pharmaceuticals, Seattle, USA) and 50 U/ml IL-4 (Miltenyi Biotec, Bergisch-Gladbach, Germany). Cells were fed on third and sixth day with X-VIVO-15, 1600 U/ml GM-CSF and 50 U/ml IL-4. After 1 week, immature DCs were harvested. For generation of macrophages PBMC were seeded in six-well plates at 0.5×10^7^ cells/3 ml/well in RPMI 1640 containing 1.5% autologous plasma for 60 min (37°C, 5% CO_2_). Non-adherent cells were removed and the remaining cells were further cultured in X-VIVO-15 with 800 U/ml GM-CSF. Macrophages were harvested after 6 days. All cell preparations were checked as to cell surface markers by flow cytometry, and preparations that were of low purity (<75%) were excluded.

### Flow Cytometric Analysis

For phenotyping, monocytes, dendritic cells and macrophages were carefully washed in cold PBS with 0.1% BSA and incubated for 20 min at 4°C with each mAB (5 µg/ml). After washing with cold PBS/BSA the cells were analyzed by flow cytometry (FACSCalibur, CellQuest software, BD Biosciences, Mountain View, CA) with data being collected on 10^4^ viable cells. The following antibodies (mAB) were used for immunofluorescence staining. Mouse IgG: CD14-PE (TÜK4), CD3-PE (BW264/56), CD19-PE (LT19; Miltenyi Biotec, Bergisch-Gladbach, Germany), CD80-PE (B7-1), CD86-PE (B7-2), HLA-DR-FITC (eBioscience, San Diego, USA); and mouse-specific isotypes, IgG-PE (S43.10; Miltenyi Biotec, Bergisch-Gladbach, Germany) and IgG-FITC (679.1Mc7; Beckman Coulter, Fullerton, USA).

### Quantification of Apoptosis

Apoptosis was measured by subG1 assay. After treatment with TMZ, pretreated or not pretreated with *O* ^6^-benzylguanine, monocytes, DCs and macrophages were washed in PBS, fixed in 70% ethanol for a minimum of 30 min at −20°C. DNA in the cells was stained with propidium iodide (16.5 µg/ml) in PBS after RNase (0.03 µg/ml) digestion. For each sample 10^4^ cells were analyzed on a FACS Calibur (Becton Dickinson). The number of apoptotic cells per sample was calculated using the computer program WinMDI 2.9 (Joseph Trotter). The cells were treated with 0.5 µM of the PARP inhibitor olaparib (AZD2281, Selleckchem) 1 h before TMZ treatment [12]. The samples were analyzed and calculated as described above on a FACS Canto (Becton Dickinson).

### Immunostaining of Cells

Prior to immunostaining cells were fixed with 4% paraformaldehyde (1×PBS, 4% paraformaldehyde) for 20 min. Fixed cells were permeabilized with 0.5% Triton-X100/PBS for 10 min. Blocking was done in PBST (1×PBS, 0.1% Tween 20) containing 4% BSA for 10 min at room temperature. Antibodies were diluted in PBST containing 2% BSA. Between application of primary and secondary antibodies washing steps were performed in PBST for 15 min. Incubations with all antibodies were performed for one hour at room temperature. Phosphorylated H2AX (ser139, γH2AX) was detected with monoclonal mouse anti-phoshpo-γH2AX (Ser 139) antibody (Millipore, Billerica MA, USA) and then with goat anti-mouse antibody F(ab’)_2_ fragment conjugated with AlexaFluor488 (Invitrogen, Oregon, USA). Nuclei were counterstained using 1 µM ToPro3 (Invitrogen, Oregon, USA), staining ocuured for 15 min in the dark; preparations were mounted in antifade medium and sealed with nail polish. The cells were treated 1 h with 0.5 µM olaparib in X-VIVO-15 and afterwards for 10 min with 10 mM H_2_O_2_ (Roth) in PBS. After fixation with methanol/acetic acid (3∶1) for 5 min, the cells were stained with a PAR (poly ADP-ribose) mouse antibody (kind gift of Dr. Bürkle, Konstanz) and with goat anti-mouse antibody conjugated with AlexaFluor488 (Invitrogen) in 5% milk powder in PBS with 0,05% Tween 20 [39]. For the CD14^+^ staining, cells were fixed with methanol, aceton and paraformaldehyde, blocked with normal goat serum (Invitrogen) and incubated with CD14 mouse monoclonal antibody (Abcam) and then with Cy^TM^3-conjugated AffiniPure goat anti-mouse IgG (H+L) (Jackson ImmunoResearch) in PBS with 1% BSA. The cells were analyzed with LSM710 (Carl Zeiss, Oberkochen, Germany).

### *In vitro* BER-assay

BER efficiency after treatment with temozolomide in monocytes was measured as described [19], [40].

### Preparation of Whole Cell Extracts and Western Blot Analysis

Monocytes, DCs and macrophages were harvested, washed once with ice-cold PBS, and lysed on ice in an appropriate amount of lysis buffer containing 50 mM Tris-HCl (pH 7.5), 250 mM NaCl, 1 mM EDTA, 0.1% Triton X-100, 2 µg/ml aprotinin, 2 µg/ml leupeptin, 1 µg/ml pepstatin and 97 µg/ml PMSF. After 30 min incubation, lysates were centrifuged at 13.000 *g and* 4°C for 20 min and the supernatant was recovered. The protein concentration was determined according to Bradford [41]. Cell extract (30 µg) was separated on a 10% or 7.5% SDS polyacrylamide gel at 100 V and was blotted onto a nitrocellulose membrane for 1 h at 300 mA using buffer composed of 25 mmol/L Tris-HCl, 86 mmol/L glycine, and 20% methanol. The antibodies used were pATM _Ser1981_, p-γH2AX (Ser 139) (Millipore, Billerica MA, USA), pChk1_ Ser317_ (Bethyl, Montgomery TX, USA), pChk2 _Thr68_ (Epitomics, Burlingame CA, USA), p53 (Dianova, Hamburg, Germany), pATR_ Ser428_, Chk1, Chk2, XIAP, Cleaved Caspase-8, Cleaved Caspase-3, Cleaved caspase-7 (Cell signaling, Danvers, MA USA), XRCC1 (Abcam, Cambridge, UK), ligase IIIα, Poly(ADP-ribose) polymerase (PARP), FasL, Bcl-2 (BD Biosciences), FasR, Bax and ERK2 (Santa Cruz Biotechnology, Heidelberg, Germany) as protein loading control.

### Preparation of RNA, Semi-quantitative RT-PCR and Real-time RT-PCR

Total RNA was isolated from cells using the RNA II Isolation Kit from Machery and Nagel. 1 µg RNA was transcribed into cDNA using the Reverse-iT 1st Strand Synthesis Kit (ABgene, Surrey, UK). Primer sequences used for PCR were as follows: *fasR* (up, 5′-AAGGGATTGGAATTGAGGAAGACTG-3′; low, 5′-GTGGAATTGGCAAAAGAAGAAGACA-3′) and *gapdh* (up, 5′-CCCCTCTGGAAAGCTGTGGCGTGAT-3′; low, 5′-GGTGGAAGAGTGGGAGTTGCTGTTGA-3′), which was used as loading control. Real-time PCR was performed using the SensiMix Plus SYBR & Fluorescein Kit (Bioline) and the MyIQ real-time PCR cycler (BioRad). Primer sequences used for real-time RT-PCR were as follows: *fasR* (up, 5′-TTATCTGATGTTGACTTGAGTAA-3′; low, 5′-GGCTTCATTGACACCATT-3′) and ß-Actin (up, 5′-TGGCATCCACGAAACTACC-3′; low, 5′-GTGTTGGCGTACAGGTCTT-3′), which was used as loading control.
